# Properties and Applications of Nanoparticles and Nanomaterials

**DOI:** 10.3390/ijms26020765

**Published:** 2025-01-17

**Authors:** Xiaogang Li

**Affiliations:** Institute of Nuclear and New Energy Technology, Tsinghua University, Beijing 100084, China; xiaogangli@mail.tsinghua.edu.cn

Nanomaterials have rapidly developed and attention surrounding their use has increased in recent years. The emergence of various nanomaterials, i.e., nanoparticles, nano-grained alloys, and gradient nanostructures, is expected to make it possible for materials with super or very special properties to be applied in unusual practical contexts. There is a wide range of applications for nanomaterials in biochemistry or molecular medicine, fuel cells or metal–ion batteries, and flexible electronics, as well in various components related to energy. The physical and chemical properties of nanostructures are determined by their chemical composition and structure and are also affected by the formation process, which is critical for their reliability and their use in practical applications.

The purpose of this Special Issue is to provide a research forum to report on the structure, properties, processing, and applications of nanoparticles and nanomaterials to explore more possibilities to address intractable challenges. Topics of interest include, but are not limited to, the studies mentioned above. Other relevant studies, such as the design of novel nanostructures or the modification of nanoparticles, have also been considered. The contributors to this Special Issue, comprising outstanding scholars and industrial researchers, have put forward cutting-edge and in-depth scientific views to help our colleagues deepen their understanding of the problems facing nanoparticles and nanomaterials and to address these intractable challenges.

Jeon et al. contributed a paper entitled “The Induction of Combined Hyperthermal Ablation Effect of Irreversible Electroporation with Polydopamine Nanoparticle-Coated Electrodes”, which introduces a more functionalized approach to irreversible electroporation for cancer ablation treatment, incorporating polydopamine nanoparticles into the system. It was found that the polymeric coating of dopamine nanoparticles coating the irreversible electroporation electrode induces an additional hyperthermal ablation effect alongside the electroporation effect, as validated in both of the outlined in vitro and in vivo assays.

Wang et al. contributed a paper entitled “Novel Chiral Self-Assembled Nano-Fluorescence Materials with AIE Characteristics for Specific Enantioselective Recognition of L-Lysine”, in which two aggregation-induced emission chiral fluorescent materials were synthesized. The two materials showed the significant enantioselective recognition of lysine, demonstrating their potential as novel chiral fluorescent probes, and a novel approach to the enantioselective recognition of lysine was presented.

Abdulla II et al. contributed a paper entitled “Unlocking the Luminescent Potential of Fish-Scale-Derived Carbon Nanoparticles for Multicolor Conversion”, which introduces a novel approach to addressing environmental issues by creating fish scale carbon nanoparticles from discarded fish scales. It was found that the luminescence mechanism is influenced by factors such as cross-linking emissions, aggregation-induced emissions and noncovalent interactions, resulting in concentration-dependent fluorescence and customizable emission colors, which suggests that fish scale carbon nanoparticles have significant potential for various applications.

Mutukwa et al. contributed a paper entitled “Optimisation, Synthesis, and Characterisation of ZnO Nanoparticles Using *Leonotis ocymifolia* (*L. ocymifolia*) Leaf Extracts for Antibacterial and Photodegradation Applications”, which reports the use of a plant indigenous to eastern and southern Africa that has not been utilized for the synthesis of nanoparticles. The work focused on utilizing this underused African plant to produce nanoparticles for photocatalysis and antibacterial applications in a green and sustainable way, and it was found that the plant had the potential to produce nanoparticles for different applications and performed better than chemically synthesized nanoparticles.

Alotaibi et al. contributed a paper entitled “Tuning the Electronic Properties of CumAgn Bimetallic Clusters for Enhanced CO_2_ Activation”, in which the electronic properties and CO_2_-adsorption mechanism of Cu_m_Ag_n_ bimetallic clusters were systematically studied using density functional theory. The paper emphasized that the Cu_4_Ag_1_ cluster could be a promising candidate for effective CO_2_ activation, which might pave the way for the design of cost-effective and effective catalysts for CO_2_ reductions.

Thepphankulngarm et al. contributed a paper entitled “Nanotechnology-Driven Delivery of Caffeine Using Ultradeformable Liposomes-Coated Hollow Mesoporous Silica Nanoparticles for Enhanced Follicular Delivery and Treatment of Androgenetic Alopecia”, which developed caffeine-loaded hollow mesoporous silica nanoparticles coated with ultradeformable liposomes (ULp-Caf@HMSNs) to enhance caffeine delivery to hair follicles. ULp-Caf@HMSNs improved cell viability compared to pure caffeine or caffeine-loaded HMSNs and effectively reduced ROS levels in dihydrotestosterone-damaged human follicle dermal papilla cells, suggesting it is a promising alternative to minoxidil for promoting hair follicle growth and reducing hair loss without increasing oxidative stress.

Pavlova et al. contributed a paper entitled “Self-Assembled Gold Nanoparticles as Reusable SERS Substrates for Polyphenolic Compound Detection”, which proposes a facile and inexpensive route to fabricate enhanced substrates for surface-enhanced Raman spectroscopy, with high reproducibility through the self-assembly of gold nanoparticles and the developed “aqua-print” process. The work addresses pivotal challenges in advancing surface-enhanced Raman spectroscopy.

Fang et al. contributed a paper entitled “A Rod-like Bi_2_O_3_ Photocatalyst Derived from Bi-Based MOFs for the Efficient Adsorption and Catalytic Reduction of Cr(VI)”, in which an optimal precursor of Bi-based MOFs was identified using different solvents, and rod-like Bi_2_O_3_ materials were derived via the in situ oxidation of Bi atoms in the precursor. The prepared materials showed remarkable stability after three consecutive photocatalytic cycles, providing a new perspective for the design of novel photocatalysts that can facilitate the removal of Cr(VI) from water.

This Special Issue highlights these novel approaches and views, and is committed to the further effective application of nanoparticles and nanomaterials. We sincerely thank the authors who submitted manuscripts to our Special Issue, and extend our sincere gratitude to the readers. We hope that our Special Issue will serve as a platform for dialog among scientists in the field of nanoparticles and nanomaterials. In addition, in view of our continued intake of high-quality articles on this topic, we have opened the Special Issue “Properties and Applications of Nanoparticles and Nanomaterials: 2nd Edition”; the submission address is https://www.mdpi.com/journal/ijms/special_issues/Y34FV4QW2H (accessed on 14 January 2025). The topics of interest include, but are not limited to, the structure, properties, processing, and applications of nanoparticles and nanomaterials, the design of novel nanostructures, and the modification of nanoparticles. Research articles and reviews in this area of study are welcome.

